# Deep Learning Techniques to Characterize the *RPS28P7* Pseudogene and the *Metazoa*-*SRP* Gene as Drug Potential Targets in Pancreatic Cancer Patients

**DOI:** 10.3390/biomedicines12020395

**Published:** 2024-02-08

**Authors:** Iván Salgado, Ernesto Prado Montes de Oca, Isaac Chairez, Luis Figueroa-Yáñez, Alejandro Pereira-Santana, Andrés Rivera Chávez, Jesús Bernardino Velázquez-Fernandez, Teresa Alvarado Parra, Adriana Vallejo

**Affiliations:** 1 Medical Robotics and Biosignals Laboratory, Centro de Innovación y Desarrollo Tecnológico en Cómputo, Instituto Politécnico Nacional (IPN), Mexico City 07700, Mexico; isalgador@ipn.mx; 2 Regulatory SNPs Laboratory, Personalized Medicine National Laboratory (LAMPER), Guadalajara Unit, Medical and Pharmaceutical Biotechnology Department, Research Center in Technology and Design Assistance of Jalisco State (CIATEJ), National Council of Science and Technology (CONACYT), Guadalajara 44270, Jalisco, Mexico; avallejo@ciate.mx (A.R.C.); demariterealvarado@gmail.com (T.A.P.); 3 Tecnologico de Monterrey, Institute of Advanced Materials for Sustainable Manufacturing, Monterrey 64849, Jalisco, Mexico; isaac.chairez@tec.mx; 4 Industrial Biotechnology Unit, Center for Research and Assistance in Technology and Design of the State of Jalisco, A.C. (CIATEJ), Guadalajara 44270, Jalisco, Mexico; lfigueroa@ciatej.mx (L.F.-Y.); apereira@ciatej.mx (A.P.-S.); 5 Consejo Nacional de Ciencia y Tecnología (CONACYT), Av. Insurgentes sur 1582, Alcaldía Benito Juárez, Mexico City 03940, Mexico; jbvelazquez@ciatej.mx; 6 Unidad de Biotecnología Médica y Farmacéutica, CONACYT-Centro de Investigación y Asistencia en Tecnologia y Diseño del Estado de Jalisco AC, Av. Normalistas 800, Colinas de la Normal, Guadalajara 44270, Jalisco, Mexico

**Keywords:** deep learning, pancreatic cancer, lethality, biomarkers, gene copy number, gene expression

## Abstract

The molecular explanation about why some pancreatic cancer (PaCa) patients die early and others die later is poorly understood. This study aimed to discover potential novel markers and drug targets that could be useful to stratify and extend expected survival in prospective early-death patients. We deployed a deep learning algorithm and analyzed the gene copy number, gene expression, and protein expression data of death versus alive PaCa patients from the GDC cohort. The genes with higher relative amplification (copy number >4 times in the dead compared with the alive group) were *EWSR1*, *FLT3*, *GPC3*, *HIF1A*, *HLF*, and *MEN1*. The most highly up-regulated genes (>8.5-fold change) in the death group were *RPL30*, *RPL37*, *RPS28P7*, *RPS11*, *Metazoa*_*SRP*, *CAPNS1*, *FN1*, *H3*−*3B*, *LCN2*, and *OAZ1*. None of their corresponding proteins were up or down-regulated in the death group. The mRNA of the *RPS28P7* pseudogene could act as ceRNA sponging the miRNA that was originally directed to the parental gene *RPS28*. We propose *RPS28P7* mRNA as the most druggable target that can be modulated with small molecules or the RNA technology approach. These markers could be added as criteria to patient stratification in future PaCa drug trials, but further validation in the target populations is encouraged.

## 1. Introduction

The success rate in cancer drug development is among the lowest of all therapeutic areas [1]. This could be explained because the incomplete understanding of the pathophysiology of complex diseases is one of the significant hurdles for target identification. Pancreatic cancer (PaCa) is estimated at 62,210 cases distributed almost equally between men and women, with an estimated death toll of 49,830 annually [2]. Pancreatic cancer is the fourth leading cause of cancer deaths in the United States and is projected to become the second deadliest cancer by 2030 [3]. Even when it is less frequent than other types of cancer, pancreatic cancer is the most lethal cancer with a 5-year survival rate of less than 9% . This survival rate has not been changed significantly in a follow-up study of 20 years in seven high-income countries [4]. It is worth mentioning that there are marked differences among PaCa patients in survival times. For those patients with metastatic cancer (50–60% of cases), the survival time ranges from 3–9 months. In the other extreme, those patients with resectable tumors can survive from 20.1 to 23.6 months [5,6]. These differences could be partially determined using variants in their genomes as copy number variants (CNVs), as well as altered gene and/or protein expression.

Pancreatic cancer is located mainly in exocrine tissue and it develops in approximately 95% of the cells that correspond to the tissue of the glands and the duct of the pancreas named pancreatic ductal adenocarcinoma (PDAC). The function of duct glands is characterized by secreting enzymes that serve to digest food in the duodenum, such as phospholipases. At the same time, the other 5% develops in endocrine cells, where the producers of the hormones (insulin and glucagon) belonging to the group of cells are found in the pancreatic islets, known as islets of Langerhans. For pancreatic cancer, in portal GDC cancer, 2753 cases have been reported, of which 13,116 genes have been identified with 34,103 somatic mutations, distributed mainly in adenomas-adenocarcinomas with 59.1% , ductal-lobular with 27.9% and epithelial neoplasms with 7.8% , of which the distribution by gender is 47% women and 53% men. Five of the first genes found among reported cases were *KRAS*, *TP53*, *SMAD4*, *CDKN2A*, and *TTN*. The *KRAS* gene has been reported to be associated with the absence of *TP53* in chronic inflammation [7]. Also, it has been reported that lipid droplets (LDs) are implicated in reprogramming tumor cell metabolism as well as the invasion and migration of pancreatic cancer cells, a bioinformatic method searching for LDs-associated markers which led to the identification of 39 up- or down-regulated genes associated with pancreatic cancer. Among these, nine genes (*CAV2, CIDEC, HILPDA, HSD17B11, NCEH1, RAB5A, SQLE, BSCL2*, and *FITM1*) were associated with overall survival [3].

In addition, *ITGA2*, *LAMB3*, and *LAMC2* gene expression were proposed as markers of early overall survival [8]. Another group reported *GRAP2, ICAM3*, and *A2ML1* as the most relevant genes in The Cancer Genome Atlas database [9]. Nevertheless, with these advances, there is a need to find and validate confident survival drug targets using a more integrative deep-learning approach, including copy number variants of genes (DNA), RNAm levels, and protein expression levels. To fulfill the promises of precision medicine, the discovery of biomarkers that could predict extended survival, as well as the discovery of novel drug targets, will be key to increasing Quality Adjusted Life Years (QALYs) of cancer patients [10].

Although high-throughput sequencing techniques produce more reliable and comprehensive lectures of properties at the level of different biomolecules, they are limited by the functional role of each molecular type in biological systems. The analysis obtained with single omics data can only study the correlation between a single molecular level and disease. This is not enough when it is necessary to comprehend specific biological phenomena. The integration and analysis of multi-omics data can compensate for missing or unreliable information in single omics data, which is helpful to explore the occurrence and development mechanism of diseases more systematically and to provide a new idea for the early diagnosis of diseases. The amount of information regarding the application of multi-omics analysis is challenging to handle via classical statistics. This directly results from high noise, high multidimensionality, and multidimensional heterogeneity. Advanced artificial intelligence techniques can compensate for the previously mentioned problems. Artificial neural networks (ANNs) have been extensively applied in non-linear identification and the classification of complex functions.

ANNs are software tools extensively used for characterizing the intrinsic relationship between input and output datasets that can be used for biomarker identification and classification [11]. The main justification of an ANN application on this complex task is their ability to deal with non-linearity within the biology datasets and the presence of uncertainties in their functional relationship. These features enable ANNs to address the identification via non-parametric modeling of patterns hidden in the data [12]. Usually, ANN structure considers a weighted, directed graph, interconnecting artificial neurons (i.e., processing nodes) organized in layers with active synapses (i.e., links) represented by a value (i.e., weight) that transmits information (i.e., signals) from one node at a preceding layer to some other nodes in the next operative layer. However, traditional feed-forward ANNs may not be efficient in creating well-defined connections among the input data if they are firmly related, such as omic information. In this paradigm, deep learning tools offer new ANN topologies that can consider a functional interdependence in the input data that explains biological functionalities [13].

A deep learning process operates as a classifier using the collected information for pancreas cancer prevalence. This study considered the application of the Long Short-Term-Memory (LSTM), a class of recurrent artificial neural networks (ANN). LSTM represents the most extended ANN architecture applied in time-dependent signal regressions and classification tasks. These ANN structures preserve long- and short-term (over the samples entering the classification process) dependencies, representing a significant benefit compared with traditional Recurrent Neural Networks considering the nature of bioinformatics information. LSTM uses a relatively well-characterized method for developing the network training. The algorithm mentioned is called Backpropagation Through Time (BPTT), which updates the parameters needed to create the relationship between the input and output sets. LSTM processes the input information flow using internal connections from and to internal state cells. These internal connections reduce the computational complexity during training and create a state that acts like a long-term memory [U-LSTM]. The LSTM cells have been useful in several tasks for classifying the bioinformatics data. Indeed, modifications such as gate recurrent units, bidirectional-LSTM, and variants with diverse internal connections have been proposed to consider the nature of the data corresponding to cancer information. Even though Deep Operator Networks (DeepONets) and the Fourier Neural Operator (FNO) and their various variants, among others, could be other options, the current selection of LSTM showed significant outcomes as is confirmed in the Results section. This fact justifies the selection of these particular artificial networks.

Given the non-parametric modeling abilities of LSTM, this study aims to design a deep learning algorithm to estimate a functional relationship between the gene copy number, gene expression, and proteome data and the vital status of patients in the dead group versus patients in the alive group in the cohort of pancreatic cancer (PaCa) patients from the GDC portal. Two genes with a higher relative amplification and higher protein expression were *HIF1A* and MEN1. The up-regulated genes (>8.5-fold change) in the dead group were *RPS28P7*, of which mRNA could act as ceRNA to miRNAs targeted to parental gene *RPS28*. *LCN2* could act as a marker for cachexia in PaCa. The discovery of these potential novel markers and drug targets could be helpful to stratify and extend the expected survival in prospective dead group patients, respectively. However, further in vitro/in vivo validation is needed.

## 2. Materials and Methods

### 2.1. TCGA Set and Collected Information about Patients with Pancreatic Cancer

The used multiomics HCC data come from the TCGA portal: https://tcga-data.nci.nih.gov/tcga/, accessed on 30 January 2023. The software TCGA—Assembler (v1.0.3; see the work in [14] ) runs on R compiler https://www.r-project.org (accessed on 30 January 2023.) to obtain the samples corresponding to the pancreas cancer cases. The obtained samples are 360 samples with DNA sequencing (DNA-Seq) data (UNC IlluminaHiSeq_DNASeqV2; Level 3), RNA sequencing (RNA-Seq) data (UNC IlluminaHiSeq_RNASeqV2; Level 3), miRNA sequencing (miRNA-Seq) data (BCGSC IlluminaHiSeq_miRNASeq; Level 3), and DNA methylation data (JHU-USC HumanMethylation450; Level 3). The relative relevance of the information contained in the acquired data has not been previously defined. Therefore, a preliminary screening for the collected information led to the selection of the following elements as components of the input matrix:

1. ASCAT DNA-Seq analysis pipeline
  (a) The weighted median of the strand copy numbers
  (b) The greater strand copy number of the two DNA strands (copy number segment files only).
  (c) The smaller strand copy number of the two DNA strands (copy number segment files only).
2. RNA-seq
  (a) The upper-quartile FPKM (FPKM-UQ) is a modified FPKM calculation in which the protein-coding gene in the 75th percentile position is substituted for the sequencing quantity.
3. miRNA sequences
  (a) miRNA read count and normalized count in reads-per-million.
  (b) Isoform information (coordinates of the isoform and the type of region it constitutes within the full miRNA transcript).
4. Methylation
  (a) Ratio between the methylated array intensity and total array intensity
5. Peptide/protein counts
  (a) The unique ID for the target site that the antigen binds to protein_expression. Relative levels of protein expression–interpolation of each dilution curve to the “standard curve” (supercurve) of the slide (antibody).

Therefore, nine components represent the largest number of elements entering the ANN, which is intended to obtain the relationship between the selected input data and the vital status of the patients who suffer from pancreatic cancer. No normalization process was considered for the input data. The data were obtained using different manifests that ran in Matlab 2022a. These manifests correspond to cases characterized by pancreas cancer, with vital status at either alive or deceased. All downloaded files were pre-processed according to the following strategy using a preliminary data import process that automatically detected the class of information in the file and associated it with the input spot in the deep learning program. According to the work proposed in [15], CpG islands (CGIs) are, on average, 1000 base pairs (bp) long and show an elevated G+C base composition, little CpG depletion, and frequent absence of DNA methylation. Three preliminary stages were performed to handle the missing values (preprocessing data information). In the first stage, the biological features (genes/miRNAs, among others) were removed if zero value appeared in patients above 20%. The incomplete samples were eliminated from the analysis if missing across more than 20% features. In the second stage, the input function from the R impute package allowed us to fill out previously missed values. In the last stage, we removed null input features with zero values across all the input samples.

### 2.2. Design of Artificial Intelligence Characterization of Vital Status

**Figure 1:**
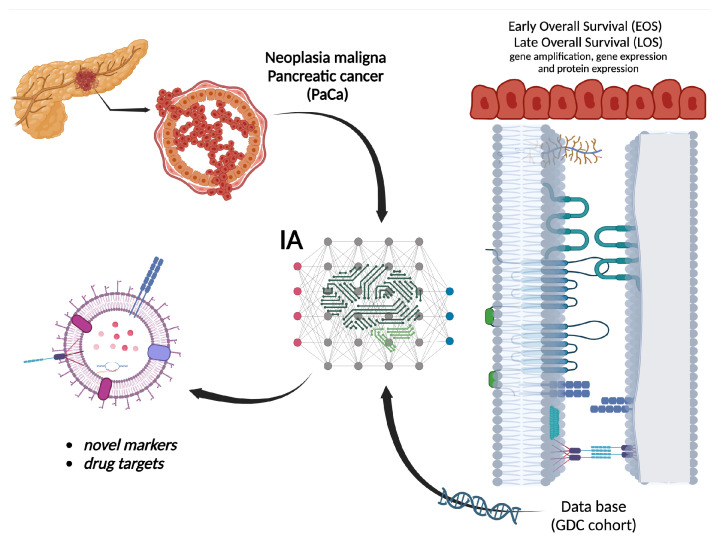
General methodology. Procedure to identify the survival expectation in patients with pancreatic cancer based on Deep Learning aiming to detect possible markers or drug targets.

The application of LSTM as the core component of a deep learning approach extracts the significant gene expression, protein distributions, miRNA, and methylation relationships with the survival of patients suffering from pancreatic cancer (Figure 1).

**Figure 2:**
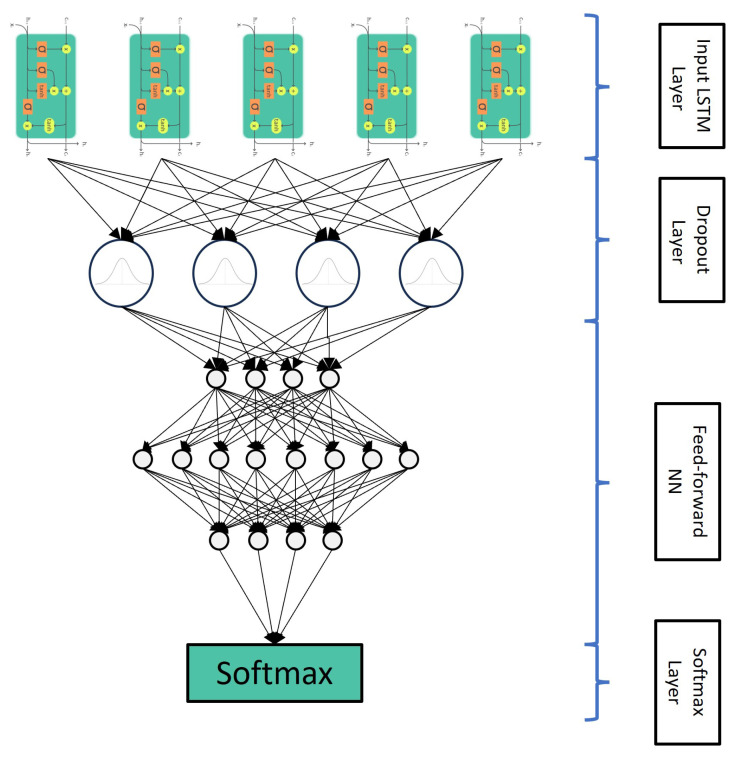
ANN Topology. The ANN has an input layer based on LSTM, a dropout layer, a feedforward neural network, and a softmax layer as the output layer.

The topology of the selected ANN operating as a classifier obeys the design presented in Figure 2. The network included four layers: a pure LSTM layer, a dropout layer, a fully connected feed-forward layer, a softmax section, and the classification result. This particular design of the ANN structure obeys traditional schemes that have been proven to work in classifying tasks of complex input–output relationships consistent with the bioinformatic information considered in this study. The LSTM layer contained 128 hidden units. This value was fixed using a progressive adaptation of the structure. The number of hidden units increased, considering the accuracy value obtained in the classification task as a criterion. This corresponded to a class of structure adaptation using a stoppage criterion in the LSTM definition.

In this case, the activation functions were selected using the following procedure: The selection of sigmoidal functions to keep a certain standard method in the topology configuration of LSTM was considered. The distribution of the sigmoidal functions was defined with parameters $P_{j}$ for the *j*- th function chosen according to a partition defined as follows $P_{j}\in \left\{P_{j,min}<P_{j,2}<...,P_{j,N-1}<P_{j,max}\right\}$ . Here, *N* is the number of functions in the distribution. The selection of the number of activation functions for each of the selected sources of omic information considered determine the largest amount of DNA, RNA, and proteins to define the size of the input vector for each LSTM in the first section of the network. To handle those entrances that do not have the largest number of these chains, zero padding was considered to homogenize the LSTM form. These LSTM memories have one output that enters the dropout layer and serves as the input to the feed-forward layer. The ANN network had five inputs for the case when all the omics information was considered in the network. The training process for the feed-forward network included the adjustment of hidden layers, yielding to a final configuration with two hidden layers with 96 and 76 activation functions, respectively.

To evaluate the relative importance of each subset of the input data (DNA-seq, RNA-seq, Methylation, miRNA-seq, and Protein), a layer formed with switching off–on activation functions is considered between the LSTM section and the dropout section. This scheme allowed for the participation of a subset in the input data to be turned on or off. This design strategy allowed us to evaluate the relative importance of each subset in the classification result and their possible combinations. This study considered the evaluation of each subset and the combination of all the inputs entering together in the proposed classifier. The dropout layer is introduced using a probability-based criterion with a drop value of 0.5 to remove over-fitting forced by the ANN. Traditionally, the fully connected feed-forward structure helps to construct the relationship between the dropped-out values and the target class corresponding to the vital status of the studied cases. This feed-forward structure has a hidden layer and an output layer, with the first having nine activation functions and the second having two outputs. The softmax layer contains two elements that define the classification outcome. The construction of the feed-forward structure with nine outputs also derived the potential construction of an autoencoder that could be further used to determine the relative importance of each input component. The selection of the parameters used in the LSTM structure considered a uniform distribution for the parameters in a pre-selected interval for each parameter in the activation function class. Therefore, this study avoids biasing the selection of the activation functions in the LSTM structure. Given the relative complexity of the LSTM structure, this study did not implement any adjustment of the parameters in the activation functions. Including adaptive parameter adjustment laws could improve the sensitivity outcomes at a marginal level but still be of significant interest. In future approaches, it is expected to consider the application of techniques such as the ones presented in [16,17,18] to observe the effect of adaptation in activation functions.

### 2.3. Training Process of Artificial Intelligence Algorithm

The availability of the vital status information in the collected database allowed us to perform a class of supervised learning. This strategy simplified the design of the classifier by considering a strategy based on transfer learning. Transfer learning is a deep learning approach in which a model trained for one task is used as a starting point for a model that performs a similar task. This scheme accelerates the adjustment of the proposed ANN.

The training of the LSTM considered the application of the traditional k-fold cross-validation. This study uses a value of k = 5 to construct the training and eventual validation of the deep learning method developed here. This strategy leads to the construction of five folders of input information. However, the disparity between the input data lengths induced the necessity of constructing asymmetric input vectors in the input signals. The data were split randomly into five folders. As usual, there are five rounds of training validation, with four folders of data serving for the training and one for validation. The proposed data partitioning assesses the robustness of the developed model by considering the variability of information contained in the input information. With the inclusion of the feed-forward structure in the proposed ANN, the weights from each layer allowed us to extract genes with a strongly propagated influence on the reduced-dimension internal encoding in the feed-forward section. Such a structure operated as an autoencoder structure that could be used to define the relative importance of each gene, RNA, protein, or other compound included in the input information.

Intending to confirm the representatives of the proposed LSTM as an effective classifier of the input information, the sensitivity analysis concerns each of the weights in the network structure. The sensitivity absolute average is calculated as the sum of the average temporal distribution of the absolute values of partial derivatives of the input–output pairs. The applicability of the sensitivity outcomes considered that each of the absolute values of the relative variations in the selected metrics for each component of the weights is larger than some predefined threshold value ( ε>0.01 ). Implementing this rule allows for the classification task based on the LSTM to be restarted until all the sensitivity values are above the selected threshold. In this reported application, there was a sequence of twelve runs until all the sensitivity outcomes satisfied the given threshold.

### 2.4. Performance Evaluation

The performance of the proposed model was evaluated using sensitivity, specificity, and accuracy. As usual, the sensitivity determines the ratio of positive samples effectively classified as true positives, i.e., the proportion of patients with the correct vital status suffering from pancreatic cancer. The specificity corresponds to the ratio of negative samples that are correctly classified as healthy, i.e., the proportion of normal individuals that are classified as healthy. The accuracy is the proportion of samples that are correctly classified. To measure the stability of the performance of the proposed model, the data is divided into training and testing data with 5-fold cross-validation. Each selected input in the database was divided according to the 5-fold cross-validation rule. Given the LSTM topology, the individual sets for each input are combined in the network, leading to an unbiased selection of information. Moreover, this strategy simplifies the inclusion of diverse input sets with different numbers of components. The effect of the input sets’ bias is voided using the early stopping condition and the sensitivity analysis.

### 2.5. Evaluation Metrics

The considered evaluation metrics reflect the accuracy of vital status prediction in the data subsets identified. The three sets of evaluation metrics are included in the following: Concordance index. The concordance index (C-index) corresponds to the fraction of all pairs of individuals whose predicted vital status is correctly ordered based on Harrell C-Statistics. The selected C-index score near 0.70 defines a good model, whereas a score near 0.50 implies a random background. A Cox-PH model using the training set (cluster labels and the vital status data) was proposed to estimate the C-index. Hence, the survival status is predicted using the labels of the test/confirmation set. We calculated the C-index using the function of the concordance index in Matlab. The calculus of the C-index used multiple clinical features; a Cox-PH using the glmnet package instead was proposed. We considered performing penalization using ridge regression instead of the default Lasso penalization.

Log-rank P value of Cox-PH regression. The Kaplan–Meier vital status curves were developed using two risk groups. The log-rank P value of the vital status difference was also estimated. The Cox-PH model for vital status analysis was also considered. Brier score. This score function measures the accuracy of probabilistic prediction. In vital status analysis, the Brier score measures the mean difference between the observed and estimated vital status beyond a certain time. This score ranges between 0 and 1 and a larger score indicates higher inaccuracy.

## 3. Results

### 3.1. Deep-Learning Algorithm

From the *TCGA PAAD*, *CPTAC-3*, and *HCC* projects, we obtained 1666 files that had integrated DNA-Seq, RNA-Seq, DNA methylation, miRNA-Seq, and proteome data. The data were processed for these samples as described in the “Materials and Methods” section. They obtained genes (DNA-seq) from RNA-Seq, methylation, and miRNAs from miRNA-Seq, as well as proteome data as the input features, as shown in Table 1.

Table 2 compares the fundamental evaluations of accuracy obtained with all the analyses based on applying the developed ANN for the proposed subsets described in the Materials and Methods section. This table shows the processing time and the number of flops required to obtain the calculus of the suggested ANN-based classifier.

Table 3 includes the results corresponding to the confusion matrix obtained by evaluating the actual for the predicted condition. These results confirmed that the proposed network effectively predicts the relationship between actual and predicted outcomes.

**Figure 3:**
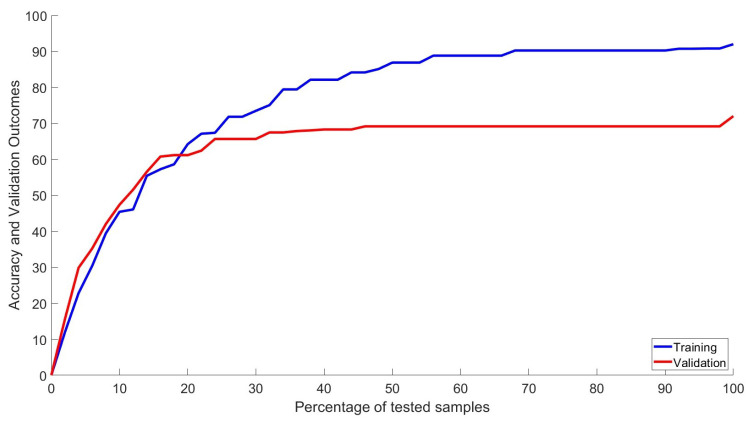
Classification performance. Receiver operating characteristic curve of the classification process of the multiomics information.

Table 4 exhibits the robustness of performance evaluation using the reproducibility and forecast indices related to the mean accuracy value for the 5-fold cross-validation, the concordance index (c-index), and the Brier score. C-index is a standard way of evaluating forecasting models’ performance in the presence of censored data. In this case, the percentage of the censored data corresponded to the same 20% used for the 5-fold cross-validation method. As a complement, the Brier score allows for estimating the accuracy of forecasting methods using probability-based predictions. Even though the sequenced information does not fully satisfy a standard probabilistic distribution, this score still offers trustful information corresponding to the quality of forecaster information based on the proposed neural network-based model. Notice that the reported information is sufficient to characterize the obtained results and does not enforce an overfitted classifier. Notice that the 5-fold and three copy numbers could be relevant as markers for the dead group/alive group. However, due to the manuscript’s topic, which is focused on drug targets and not biomarkers, we include just the top 10 drug targets with the highest DEGs. The study of the performance evaluation presented in this study considered the application of the receiver operating characteristic curve or ROC curve. This plot shows the classification ability of the proposed network system as its discrimination threshold is varied. The obtained result shows when the entire set of inputs enters the composite network based on the combination of LSTM structures. Figure 3 shows the evolution of the training and validation outcomes as functions of the percentage of the entire data considered in the study. The behavior of these results appears to correspond to the regular evolution of classifiers based on the class of recurrent networks used in this study.

The training and the validation were performed several times. The differences between training and validation could be seen as a potential overfitting. Nevertheless, this difference establishes the complexity of the relationship between the omics data and pancreatic cancer’s long-term survival. Also, the validation was evaluated several times using different information subsets. The reported values for training and validation are those obtained as an average, which helps us better justify the application of the machine learning methodology.

### 3.2. Gene Amplification

The genes with higher relative amplification (>4 times in the dead group compared with the alive group) are shown in Table 5.

### 3.3. Gene Expression

**Figure 4:**
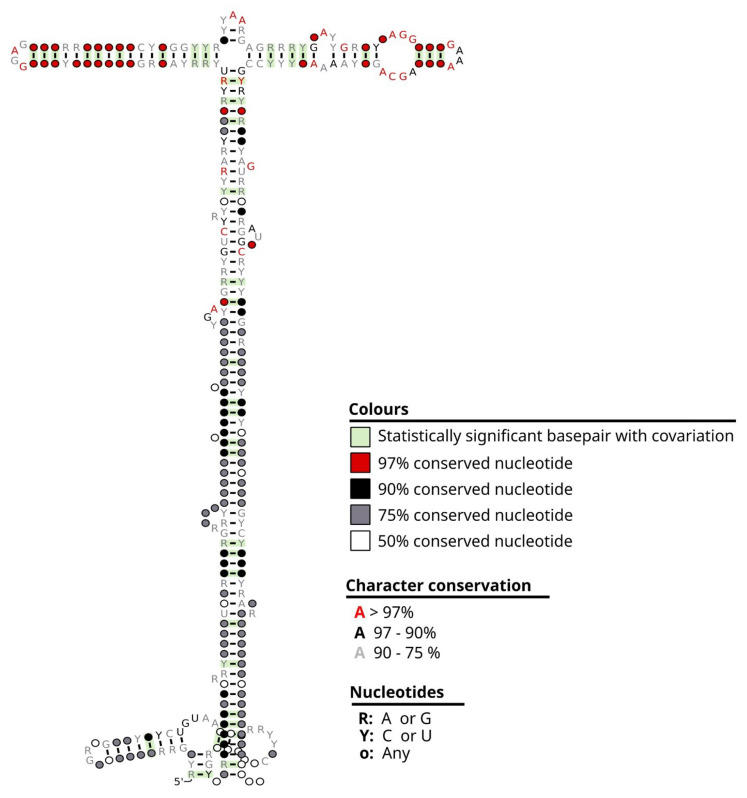
Metazoa_SRP RNA representation. Representation of the secondary structure of the non-coding Metazoa_SRP RNA (signal recognition particle RNA; RF00017) part of the signal recognition particle (SRP) ribonucleoprotein complex. The representation was depicted by Rscape [28], using the alignment of 91 metazoan species from the Rfam database [14].

Differentially expressed genes (DEGs) of the dead group ( n=310 ) compared to the alive group ( n=896 ) are shown in Table 6. As reported in the literature, the correlation of gene expression and protein expression varies greatly, e.g., from 0.07 to 0.91 in [27]. For that reason, both markers (mRNA and protein) were analyzed independently. Regarding gene expression, the only report we found in pancreatic cancer lethality was Bai et al., 2021. None of the nine genes reported in [3] associated with survival in lipid droplets (LDs) were found to be significantly up- or down-regulated in our analysis. This may be due to differences in the type of analyzed sample, as explained in the discussion section. The sequence of Metazoa_SRP RNA with its annotated mutations and 2D predicted structure is shown in Figure 4.

### 3.4. Selection of Hits as Potential Drug Targets

Since CNVs are not druggable and protein levels were not informative for a dead or alive status, we focused on those altered genes that have both the highest relative expression and novelty as PaCa markers. These genes were *RPS28P7* and *Metazoa*_*SRP*. A search in the open Targets Platform (https://platform.opentargets.org/target, accessed on 30 January 2023) and KEGG database (https://www.genome.jp/kegg/, accessed on 30 January 2023) of both genes retrieved no results. However, in the GWAS catalog (https://www.ebi.ac.uk/gwas, accessed on 30 January 2023), SNPs in *Metazoa*_*SRP* gene were previously associated with epithelial ovarian cancer, differentiated thyroid cancer, and papillary thyroid cancer, as well as with breast, colorectal, and lung cancers.

### 3.5. Protein Expression

Regarding the results of protein expression, we verify if any of their corresponding nine proteins (because one of them was the pseudogene *RPS28P7*) of Section 3.3 (gene expression) were also up-regulated in the dead group when compared with the alive group. Unfortunately, there were incomplete/null data regarding the *CAPNS, LCN2*, and *H3F3B* proteins in the GDC portal for this cohort. The averages of protein quantification for each group are shown in Table 7. Protein expression analysis revealed that none of the nine proteins correlate with their corresponding gene up-regulation and the differences among the dead and alive groups are not significant.

### 3.6. Selection of Hits as Potential Drug Targets

Due to the fact that CNVs are not druggable and protein levels were not informative for a dead or alive status, we focused on those altered genes that have both the highest relative expression and novelty as PaCa markers. These genes were *RPS28P7* and *Metazoa*_*SRP*. A search in the open Targets Platform (https://platform.opentargets.org/target, accessed on 30 January 2023) and KEGG database (https://www.genome.jp/kegg/, accessed on 30 January 2023) of both genes retrieved no results. However, in the GWAS catalog (https://www.ebi.ac.uk/gwas, accessed on 30 January 2023), SNPs in the *Metazoa*_*SRP* gene were previously associated with epithelial ovarian cancer, differentiated thyroid cancer, and papillary thyroid cancer, as well as with breast, colorectal, and lung cancers.

## 4. Discussion

Regarding gene amplification, *EWSR1, FLT3, GPC3, HIF1A, HLF*, and *MEN1* have been reported in CaPa and other neoplasias as well. In particular, the *HIF1A* protein induces metabolic reprogramming in the hypoxic condition of a pancreatic tumor and up-regulates multiple genes as *cyclin D1*, *Met protooncogene*, receptor Tyrosine kinase (*MET*, formerly *HGFR*), vascular endothelial growth factor A (VEGFA), carbonic anhydrase IX (CAIX), fibronectin, and glucose transporter 1 (GLUT1) [6]. Copy number variants (CNVs) are considered relevant markers independent from DEGs (differentially expressed genes). Nevertheless, the highest DEGs are included in Table 6, but none were those of the CNVs.

The *MEN1* gene encodes menin, a nuclear scaffold protein that regulates gene transcription by coordinating chromatin remodeling. Menin interacts with several transcription factors, including oncogene Jun-D, NF-kB, and Sma and Mad-related protein 3 (SMAD3). *MEN1* is considered a tumor suppressor gene [29]. *MEN1* is the most frequently mutated gene in pancreatic neuroendocrine neoplasms (pNEN) [30] and its function has also been suggested in diverse familial and sporadic tumors of endocrine origin [31]. Menin protein binds and regulates several genes, including telomerase reverse transcriptase (hTERT), Hox family genes, and the cyclin-dependent kinase inhibitor genes p27 and p18. All these genes are involved in tumor suppression or cell differentiation. Menin activates transcription by recruiting MLL to both p27 and p18 promoters and coding regions. The exact mechanisms of a tissue-specific function of menin remain to be elucidated [32]. It is worth mentioning that our results suggest that *MEN1* could also be relevant in PDAC neuroendocrine as previously reported, but is also relevant in PDAC because, in this study, all cases are patients with ductal and lobular neoplasias.

Regarding gene expression, none of the nine genes reported by Bai et al. [3] associated with survival in LDs, were found to be significantly up- or down-regulated in our analysis. This may be due to differences in the type of analyzed sample. We include just the top 10 drug targets, which are the most significant DEGs.

Calpains are heterodimeric calcium-dependent cysteine proteinases classified as calpains I and II. Both types of calpains share a light (∼30 kDa) regulatory subunit, encoded by the *CAPNS1* gene. *CAPNS1* is one out of five key prognostic autophagy-related genes in hepatocellular carcinoma [33]. In both MCF7 and MCF10AT cell lines, *CAPNS1* depletion leads to the enlargement of the stem cell compartment in breast cancer [34].

Fibronectin-1 (FN1) is a glycoprotein that interacts with other extracellular matrix proteins and cellular ligands such as integrins, fibrin, and collagen. The two most abundant proteins in the cargo of extracellular vesicles shed by macrophages in PDAC are FN1 and chitinase 3-like-1 (CHI3L1). Pirferidone inhibits FN1 and this partially reverted gemcitabine resistance [35]. Furthermore, FN1 has been identified as one out of seven hub genes in PDAC [8].

The *H3-3B* gene belongs to the so-called replacement histones because they are replication-independent and are expressed in quiescent or terminally differentiated cells. Histone H3.3 is encoded by either the identical genes H3-3A and H3-3B [36]. Mutation of these genes leads to some human cancers such as chondroblastoma, osteosarcoma, and epithelial ovarian cancer [37]. In addition, H3-3B up-regulation has been suggested as a marker for pre-metastatic colon cancer [38]. Furthermore, a circular RNA (hsa_circ_0091579) accelerated Warburg effect and tumor growth via H3-3B up-regulation by adsorbing miR-624 in hepatocellular carcinoma (HCC) [39].

Lipocalin-2 (*LCN2*), also known as NGAL, is a protein associated with neutrophil gelatinase. The 25-kD *LCN2* protein is believed to bind small lipophilic substances such as bacteria-derived lipopolysaccharide (LPS) and formylpeptides and may function as a modulator of inflammation. *LCN2* inhibits pancreatic cancer stemness via the AKT/c-jun pathway [40]. *LCN2* is an endogenous ligand of the type 4 melanocortin receptor (MC4R), a critical appetite regulator. *LCN2* levels correlate with fat and lean mass wasting and are associated with increased mortality in patients with pancreatic cancer. Taken together, these findings recently implicate *LCN2* as a pathologic mediator of appetite suppression during pancreatic cancer cachexia [41].

The metazoan signal recognition particle RNA gene (Meta-zoa_SRP) encodes ribosomal ribonucleoproteins 4.5S (also named *4.5 S*, *7SL* or *6S*). SRP recognizes the signal peptide and binds to the ribosome, halting protein synthesis. SRP also directs the fundamental movement of proteins within the cell by binding to the transmembrane pore, which allows the proteins to cross the membrane to where they are needed (https://rfam.org/family/RF00017, accessed on 30 January 2023).

Ornithine decarboxylase antizyme (*OAZ1* gene) is a potential therapeutic target in various malignant tumors because it plays relevant roles in cellular functions, including genomic stability, proliferation, differentiation, and apoptosis [42]. The enhancer-related lncRNA-mRNA pairs as prognostic biomarkers AC0-27307.2-*OAZ1* in the Basal-like subtype of breast cancer [43] and as a fusion gene. The up-regulation of the *OAZ1* gene has been demonstrated in three studies in oral squamous cell carcinoma (OSCC) [44] and chronic myeloid leukemia (CML) [42]. *OAZ1* was down-regulated in cisplatin-resistant non-small-cell lung cancer [45].

In spite of some authors considering *RPL30* as a classical reference gene for cancer research due to its stable expression [46], our results showed that the overexpression of *RPL30* is a hallmark of the dead group in PaCa, being the highest overexpressed gene in this GDC cohort (26.06-fold on average). Likewise, the *RPL30* gene has been suggested as one out of eight major genes that predict poor clinical outcomes in mucinous colorectal adenocarcinoma [47] and is also informative for lethality in medulloblastoma [48]. In addition, *RPL30* is one of seven genes whose expression levels have been proposed for diagnosing prostate cancer [49].

The *RPL37* gene is constitutively expressed even during transitions from quiescence to active cell proliferation or terminal differentiation in all tissues and all vertebrates investigated. Its specific role in cancer has not been elucidated. However, *RPL37* is one out of ten histotype-specific prognostic biomarkers for early-stage clear-cell (CCC) ovarian carcinoma [50] *RPL37*, together with two other ribosomal proteins RPL15 and RPS20 which bind to Mdm2 and activate p53. After that, each RP can down-regulate MdmX levels but via distinct pathways [51].

*RPS11* is a ribosomal protein involved in ribosome biogenesis. Its gene *RPS11* is also the host gene for U35 (SNORD35B), an intronic small nucleolar RNA (snoRNA) [52]. The *RPS11* protein is overexpressed in diverse malignancies and correlates with tumor recurrence. *RPS11* is a target of hsa-miRNA-142-3p. In non-small-cell lung cancer (NSCLC), this gene significantly impacts proliferation in all of the tested cell lines [53]. In hepatocellular carcinoma (HCC) tumors, high *RPS11* levels were associated with shorter overall survival (OS) and recurrence-free survival (RFS) of HCC patients after curative resection [54].

*RPS28P7* is a processed pseudogene (See the following webpage https://www.ensembl.org, accessed on 30 January 2023) that originated as a retrocopy of the parental gene *RPS28*. LncRNAs, or mRNA of pseudogenes (literally “false genes”), often act as sponges that bind non-coding miRNAs, thus indirectly modulating the half-life of the mRNA of the parental gene. Based on this function, these RNAs are called competitive endogenous RNAs (ceRNAs). There are at least 13 lncRNAs that act as ceRNAs in PaCa [55]. Regarding ceRNAs of pseudogenes, these contribute to oncogenesis, as the *BRAF* pseudogene does in lymphoma [56], as well as other ceRNAs in colorectal [57], breast [58], ovarian [59], and among other types of cancer (reviewed in [55,60]). Furthermore, ceRNAs mediate autophagy, chemoresistance, and metastasis [61].

Our results suggest that *RPS28P7* mRNA could regulate the RPS28 gene in this way, acting as a sponge for suppressor miRNAs originally targeted to *RPS28* mRNA. This could lead to increased *RPS28* protein expression, contributing to a poorer prognosis because in this study, *RPS28P7* was associated with an earlier overall survival (dead group) in this GDC cohort. It is worth mentioning that *RPS28* is one out of the nine up-regulated hub genes in multiple myeloma (MM) [62] and also is one out of the seven prognosis-related genes of RNA-binding proteins suggested as a prognosis panel for oral cavity squamous cell carcinoma (OCSCC) [63].

Recent technologies make it feasible to identify or design chemical matter that binds RNA as novel drug candidates [64]. One of these approaches could be helpful to develop novel small molecules that target the mRNA of the *RPS28P7* pseudogene and the misc_RNA of the *Metazoa*_*SRP* gene. In the case of *RPS28P7*, the most logical approach is to inhibit/degrade its mRNA to let miRNAs target the mRNA of the parental gene *RPS28*. In contrast, due to the fundamental role of protein translocation in the cells, the proposed approach for *Metazoa*_*SRP* is not to inhibit but to reduce its activity or the absolute numbers of its misc_RNA molecules.

Regarding protein expression, our analysis revealed that none of the nine proteins correlate with their corresponding gene up-regulation and differences among dead and alive groups, which were not significant. As reported in the literature, the correlation of gene expression and protein expression varies greatly, e.g., from 0.07 to 0.91 in [27]. Therefore, both markers (mRNAs and proteins) were analyzed independently and we expected to obtain complementary information regarding known and novel drug targets.

The main limitation of our study is that we did not quantify the days from the diagnosis to death. However, we realize that in the dead group, we found patients with up to 4 years of earlier diagnosis than the earliest diagnosis of the alive group.

The deep learning analysis we performed was based on real data from patients with pancreatic cancer. That kind of data is considered in the literature for drug development and target validation as “experiments of nature” (https://doi.org/10.1038/nrd4051, accessed on 30 January 2023). We know that the lead compounds to be developed, inspired by these results, will need to be tested and validated before further development.

## 5. Conclusions

We report for the first time that the up-regulation of the *RPS28P7* pseudogene is associated with cancer and particularly predicts lethal status in PaCa patients in PaCa. The *RPS28P7* pseudogene could act as ceRNA sponging miRNA directly to the parental gene RPS28. We propose *RPS28P7* mRNA and the misc_RNA of the *Metazoa*_*SRP* gene as potential drug targets that can be blocked/degraded and modulated respectively, with a small molecule approach, RNA editing, or another RNA technology. Regarding potential biomarkers for a dead/or alive status, our results revealed that 40% of the top 10 up-regulated genes in the lethal group are related to ribosome-associated proteins, namely *RPL30, RPL37, RPS28P7*, and *RPS11*, which are all essential during the higher demand of protein translation of the rapidly growing tumors. In addition, we propose that *MEN1* gene amplification (but not its gene or protein up-regulation as in previous reports) is also a novel marker to predict lethal status in PaCa. Also, the up-regulation of the *LCN2* gene could explain cachexia, appetite suppression, and lethal status in PaCa patients. These markers could be added as criteria to support negative or positive prognostic in future PaCa drug trials, but further validation in the target populations and age cohorts is encouraged.

The selection of the LSTM structure was obtained by applying a standard and ordered method following a segment partition scheme. However, the application of adaptive methods such as the ones presented in [16,18,65] can help us to simplify the selection of the LSTM-structure and, moreover, to improve the network-based classification performance.

The inclusion of adaptive methods to adjust the parameters is something we have not considered in this study based on the key objective pursued here. However, this is an alternative that could be explored in future studies.

## Figures and Tables

**Table 1:** Number of files used to construct the input database ^1^.

Subset	Survived	Deceased
Subset 1: DNA-seq	5064	3199
Subset 2: RNA-seq	1818	1165
Subset 3: Methylation	774	504
Subset 4: miRNA-seq	848	662
Subset 5: Protein	70	50

^1^ The available information about protein expression is unbalanced compared to the other sources of information. Here, no strategies were performed to introduce artificial balance in the data distribution, considering we could obtain conclusions using the raw information instead of introducing a misunderstanding generated from the omics information.

**Table 2:** Comparison of accuracy, process time, and number of flops in the evaluated ANN with the different configurations of input configurations.

Subset	Accuracy	Process Time (Hours)	Number of Flops
Subset 1: DNA-seq	0.92	56	$1.5\times 10^{7}$
Subset 2: RNA-seq	0.92	49	$4.6\times 10^{7}$
Subset 3: Methylation	0.81	51	$7.1\times 10^{7}$
Subset 4: miRNA-seq	0.88	67	$9.4\times 10^{7}$
Subset 5: Protein	0.80	43	$2.3\times 10^{7}$
All subsets	0.96	78	$9.5\times 10^{8}$

**Table 3:** Confusion matrix for the classification case when all the subsets were considered as inputs to the proposed ANN (AC. Actual condition, PC. Predicted condition).

		PC	
		Positive (PP)	Negative (PN)
**AC**	**Positive (PP) **	4061	203
	**Negative (PN)**	128	3071

**Table 4:** Robustness of the ANN classifier on training and test sets for all subsets case (MA. mean accuracy).

Dataset	5-Fold CV (MA)	C-Index	Brier Score
Training	92%	0.76	0.81
Tests	72%	0.67	0.75

**Table 5:** Genes with higher amplification in Early Overall Survival group compared with Late Overall Survival and their known functions.

Gen (HGNC) ^1^	Locus	Relative Amplification ^2^	Dead Group (*n* = 238) CNV Mean (Range)	Alive Group (*n* = 509) CNV Mean (Range)	Function	Other Types of Cancer
*EWSR1*	22q12.2	4.04	5.34 (2–8)	1.32 (1–2)	Its RNAm binds to RNA in poly-G and poly-U	Ewing sarcoma, neuroblastoma [19]
*FLT3*	13q12.2	4.11	5.34 (3–8)	1.30 (1–2)	Growth factor receptor on hematopoietic stem and/or progenitor cells	Acute lymphoblastic leukemia, Acute myeloid leukemia [20,21]
*GPC3*	Xq26.2	4.11	5.42 (3–8)	1.32 (1–2)	Regulate the signaling of WNTs, Hedgehogs, fibroblast growth factors, and bone morphogenetic proteins	Wilms tumor [22]
*HIF1A*	14q23.2	4.35	5.65 (3–8)	1.30 (1–2)	Essential role in cellular and systemic homeostatic responses to hypoxia.	Glioblastoma [23]
*HLF*	17q22	4.03		5.31 (3–8)	1.30 (1–2) controls apoptosis of serotonergic neurons in C. elegans	Acute myeloid leukemia [24]
*MEN1*	11q13.1	4.12	5.37 (3–8)	1.32 (1–2)	Nuclear scaffold protein that regulates gene transcription by coordinating chromatin remodeling.	Adrenal adenoma, angiofibroma, carcinoid tumor of the lung, lipoma, multiple endocrine neoplasia, parathyroid adenoma [25,26]

^1^ EWSR1 (Ewing sarcoma RNA-binding protein 1), FLT3 (FMS-related tyrosine kinase 3), *GPC3* (Glypican 3), *HIF1A* (Hypoxia-Inducible factor 1, alpha subunit), *HLF* (Hepatic Leukemia Factor), *MEN1* (Menin 1). ^2^ Relative amplification (fold-change Dead group versus Alive group).

**Table 6:** Statistical study of gene expression.

Gene	Gene Name	Locus	Fold-Change (D vs. A)
*RPL30*	Ribosomal protein L30	8q22.2	26.06
*RPS28P7*	Ribosomal protein S28 pseudogene 7	11q14.1	16.81
*Metazoa_SRP*	Metazoan signal recognition particle RNA	10p12.31	10.33
*H3F3B*	H3 histone, family 3B	17q25.1	9.77
*OAZ1*	Ornithine decarboxylase antizyme 1	19p13.3	9.52
*RPS11*	Ribosomal protein S11	19q13.33	9.18
*CAPNS1*	Calpain, small subunit 1	19q13.12	9.10
*FN1*	Fibronectin 1	2q35	8.76
*LCN2*	Lipocalin 2	9q34.11	8.66
*RPL37*	Ribosomal protein L37	5p13.1	8.53

**Table 7:** Differences in protein expression among dead group and alive group.

Protein	Dead Group Mean (95% CI)	Alive Group Mean (95% CI)	F Test (*p*-Value)	T Test (*p*-Value)
*4.5 S*	NR	NR	0.159	0.974
*CAPNS1*	NR	NR	−	−
*FN1*	0.581 (1.144 – 0.018)	0.668 (0.074–1.261)	0.742	0.737
*H3F3B*	1.2935 (0.589, 1.798)	1.487 (0.625, 1.524)	−	−
*LCN2*	NR	NR	−	−
*OAZ1*	0.487 (−0.204–1.180)	0.344 (−0.236–1.005)	0.429	0.407
*RPL30*	−0.175 (−0.466–0.114)	−0.197 (−0.461–0.065)	0.546	0.555
*RPL37*	−0.259 (−0.653–0.135)	−0.216 (−0.816–0.382)	0.003	0.623
*RPS11*	−0.175 (−0.046–0.114)	0.263 (−0.461–0.065)	0.546	0.555

## Data Availability

Data are contained within the article.
